# Epstein-Barr virus reactivation in sepsis due to community-acquired pneumonia is associated with increased morbidity and an immunosuppressed host transcriptomic endotype

**DOI:** 10.1038/s41598-020-66713-3

**Published:** 2020-06-17

**Authors:** Cyndi Goh, Katie L. Burnham, M. Azim Ansari, Mariateresa de Cesare, Tanya Golubchik, Paula Hutton, Lauren E. Overend, Emma E. Davenport, Charles J. Hinds, Rory Bowden, Julian C. Knight

**Affiliations:** 1 Wellcome Centre for Human Genetics, University of Oxford, Oxford, UK; 2 Wellcome Sanger Institute, Wellcome Genome Campus, Hinxton, UK; 3 Peter Medawar Building for Pathogen Research, University of Oxford, Oxford, UK; 4 Big Data Institute, University of Oxford, Oxford, UK; 5 Adult Intensive Care Unit, Oxford University Hospitals NHS Foundation Trust, Oxford, UK; 6 William Harvey Research Institute, Barts and The London School of Medicine, Queen Mary University, London, UK

**Keywords:** Genetics, Immunology, Microbiology, Diseases, Infectious diseases

## Abstract

Epstein-Barr virus (EBV) reactivation is common in sepsis patients but the extent and nature of this remains unresolved. We sought to determine the incidence and correlates of EBV-positivity in a large sepsis cohort. We also hypothesised that EBV reactivation would be increased in patients in whom relative immunosuppression was the major feature of their sepsis response. To identify such patients we aimed to use knowledge of sepsis response subphenotypes based on transcriptomic studies of circulating leukocytes, specifically patients with a Sepsis Response Signature endotype (SRS1) that we have previously shown to be associated with increased mortality and features of immunosuppression. We assayed EBV from the plasma of intensive care unit (ICU) patients with sepsis due to community-acquired pneumonia. In total 730 patients were evaluated by targeted metagenomics (n = 573 patients), digital droplet PCR (n = 565), or both (n = 408). We had previously analysed gene expression in peripheral blood leukocytes for a subset of individuals (n = 390). We observed a 37% incidence of EBV-positivity. EBV reactivation was associated with longer ICU stay (12.9 vs 9.2 days; p = 0.004) and increased organ failure (day 1 SOFA score 6.9 vs 5.9; p = 0.00011). EBV reactivation was associated with the relatively immunosuppressed SRS1 endotype (p = 0.014) and differential expression of a small number of biologically relevant genes. These findings are consistent with the hypothesis that viral reactivation in sepsis is a consequence of immune compromise and is associated with increasing severity of illness although further mechanistic studies are required to definitively illustrate cause and effect.

## Introduction

The recently revised definition of sepsis as the life-threatening organ dysfunction caused by a dysregulated host response to infection^1^ was motivated in part by accumulating evidence to suggest that immunosuppression is a key feature of the disease and an important contributor to morbidity and mortality^2^. Human and animal studies have demonstrated that immunosuppression in sepsis involves lymphocyte exhaustion and apoptosis, reprograming of antigen-presenting cells leading to dysfunctional immune tolerance and impaired innate immune memory^3–5^. More recently, transcriptomic studies delineating sepsis endotypes have highlighted that features of immunosuppression are important in distinguishing endotypes associated with a worse outcome^6,7^.

Our group recently described two sepsis response signatures (SRS1 and SRS2) using genome-wide gene expression analysis of peripheral blood leukocytes^6^. Individuals with an SRS1 endotype were characterised by features of immunosuppression, including endotoxin tolerance, T-cell exhaustion, and downregulation of HLA class II. Importantly, 14-day mortality was significantly higher in this group, supporting the concept that immunosuppression is an important determinant of outcome in patients with sepsis. Other groups have independently made similar observations, for example the MARS consortium have described a high risk endotype characterised by decreased expression of genes involved in key innate and adaptive immune cell functions^7^.

Alongside this, studies describing a high frequency of viral reactivation in sepsis patients support the concept that previously immunocompetent individuals can develop a varying degree of functional immunosuppression in response to severe infection^8–12^. Walton and colleagues observed that 43% of septic patients had evidence of viraemia with multiple viruses^8^, the MARS consortium described a 68% frequency of herpesvirus viraemia amongst individuals with septic shock^9^, whilst Mallet and colleagues observed a 53% frequency of herpesvirus viraemia in individuals with septic shock^12^. In both studies, Epstein-Barr virus (EBV) was the most commonly observed reactivated virus at a frequency of 32–48% in plasma^8,9,12^. Neither group observed an independent association between EBV reactivation and mortality, although the MARS consortium identified a 3.17 hazard ratio for mortality with concurrent cytomegalovirus (CMV) and EBV reactivation^9^, whilst Mallet and colleagues found that reactivation of more than one virus was associated with increased ICU mortality^12^. In addition, Walton and colleagues observed an increased incidence of fungal infections, mean SOFA score and ICU length of stay in those patients with EBV reactivation^8^.

Improved understanding of viral reactivation has the potential to enhance our understanding of the immunosuppression that is key to sepsis pathophysiology. Our aims in this study were to determine the incidence of EBV reactivation; the relationship with outcome and clinical features; and how this varied by host sepsis transcriptomic endotype, specifically whether such reactivation was more frequent in the immunocompromised SRS1 endotype. If this were the case, levels of viraemia might consequently be a useful biomarker of immunosuppression, guiding immunotherapy and enabling monitoring of disease progress and response to therapy. We chose to focus on EBV as the most commonly observed reactivated virus with a seroprevalence of over 90%^13^.

## Results

### Patient cohorts

ICU patients (n = 730) with sepsis due to community-acquired pneumonia (CAP) were recruited through the UK Genomic Advances in Sepsis (GAinS) study. EBV was assayed from plasma samples taken at one or more time points (day 1 and/or day 3 and/or day 5 of ICU admission) by two methods, targeted metagenomics (757 samples from 573 patients) and digital droplet PCR (ddPCR) (619 samples from 565 patients). Targeted metagenomics enabled characterisation of multiple reactivated viruses in plasma whilst ddPCR provided a quantitative approach focused on EBV, enabling assessment of individuals from the metagenomics group at later time points as well as additional individuals. There was an overlap of 408 patients in the application of the two methods, with a total of 730 patients evaluated overall.

### Definition of sample positivity

Samples with more than one positive droplet across the three replicates were deemed ddPCR EBV-positive (equivalent to 13 copies/ml); a sample was deemed metagenomics EBV-positive if the random forest score was greater than 0.465, as described previously^14^. For downstream analyses, a sample was considered EBV-positive if either or both the ddPCR and the metagenomic result was positive. There was 85% agreement between metagenomics and ddPCR across the samples tested.

### Serological testing indicates that EBV viraemia is secondary to reactivation

We first sought to understand the extent to which observed EBV viraemia (positivity on ddPCR) would reflect viral reactivation vs primary infection. The ddPCR methodology is highly sensitive, allowing us to evaluate samples with different viral loads. We selected plasma from 40 EBV ddPCR-positive patients to represent the full range of viral loads observed (13 to 7.3 × 10^6^ copies/ml) and tested for IgG and IgM antibodies against EBV Viral Capsid Antigen (VCA). All individuals tested (n = 40; 100%) were positive for the IgG VCA antibody and negative for IgM VCA antibody, indicating that sepsis had coincided with viral reactivation and not primary infection.

### EBV reactivation is common and increases in frequency with time after ICU admission

Combining both cohorts, a total of 1042 unique samples (730 patients) from days 1, 3 and 5 after ICU admission were evaluated by either targeted metagenomics, ddPCR, or both (Supplementary Tables 1 and 2). The overall incidence of EBV reactivation at any time point was 37% (271/730). The incidence of EBV reactivation increased significantly over time (X^2^ = 24.6; d.f.=2; p = 4.6 × 10^−6^) (Supplementary Table 3).

### Morbidity is increased in EBV-positive individuals

Clinical outcome data was compared between EBV-negative (n = 459) and EBV-positive (n = 271) individuals (Table 1); patients were considered EBV-positive if at least one sample at any time point was positive by ddPCR or targeted metagenomics. There was a trend to a higher 28-day mortality (24% vs 16%; p = 0.051) and ICU length of stay was longer (12.9 days vs 9.2 days; p = 0.0040) in the EBV-positive group. In addition, the EBV-positive group had more organ failures with higher day 1 SOFA scores (6.9 vs 5.9; p = 0.00011) and maximum SOFA scores (7.9 vs 6.7; p = 3.6 × 10^−6^). There was no association between EBV reactivation and microbiological cause of sepsis (Supplementary Table 4).

**Table 1:** Clinical characteristics and outcome data.

Characteristic	Epstein-Barr virus-negative (n = 459)	Epstein-Barr virus-positive (n = 271)	p-value
Age (years)	60.5 (17–92)	62.4 (19–91)	0.11
Male sex^	275 (60%)	141 (52%)	0.049
Charlson comorbidity index*	2.8 (0–10)	2.6 (0–9)	0.30
ICU-acquired infection^	87 (19%)	66 (24%)	0.098
Mortality (28-day)^§^	72 (16%)	64 (24%)	0.051
Intensive care unit length of stay^§^ (days)	9.2 (1–101)	12.9 (1–55)	0.0040
Sequential Organ Failure Assessment score (day 1)*	5.9 (0–17)	6.9 (1–19)	0.00011
Sequential Organ Failure Assessment score (maximum over the first 7 days of admission)*	6.7 (0–19)	7.9 (1–21)	3.6 × 10^−6^

Epstein-Barr virus-negative and Epstein-Barr virus-positive individuals are compared (total n = 730). Intensive care unit length of stay analysis only includes patients surviving to intensive care unit discharge. T-test performed except where indicated (^Chi-squared test; *Mann-Whitney U-test; ^§^Log-rank test). Summary values refer to the mean. The range of values observed is provided where relevant.

### EBV is the most common of several viruses reactivated in sepsis

Amongst the 757 samples from 573 patients analysed by targeted metagenomics, viral reactivation was observed in 26% of individuals (Table 2). The probe panel used for the targeted metagenomic approach was primarily designed to target microbiological causes of adult CAP and paediatric meningitis^14^; the list of viruses included in Table 2 represent the overlap with viruses previously described to represent reactivation in sepsis^8^. EBV was the most commonly observed virus; there was a 24% incidence of EBV reactivation. Six individuals showed simultaneous reactivation of EBV and a second virus. Plasma sampling was enriched for earlier time points (Day 1 = 302; Day 3 = 284; Day 5 = 171).

**Table 2:** Incidence of viral reactivation in sepsis patients analysed by targeted metagenomics.

Other reactivated virus	None	Herpes simplex virus	Cytomegalovirus	Human herpes virus 6	JC virus
Epstein-Barr Virus negative (n = 438)	422 (74%)	5 (0.9%)	1 (0.2%)	8 (1.4%)	2 (0.3%)
Epstein-Barr Virus positive (n = 135)	129 (23%)	4 (0.7%)	1 (0.2%)	0 (0%)	1 (0.2%)

A total of 573 patients were analysed. Six patients demonstrated coreactivation with Epstein-Barr Virus and a second virus.

### EBV reactivation is associated with the immunosuppressed SRS1 endotype

**Figure 1:**
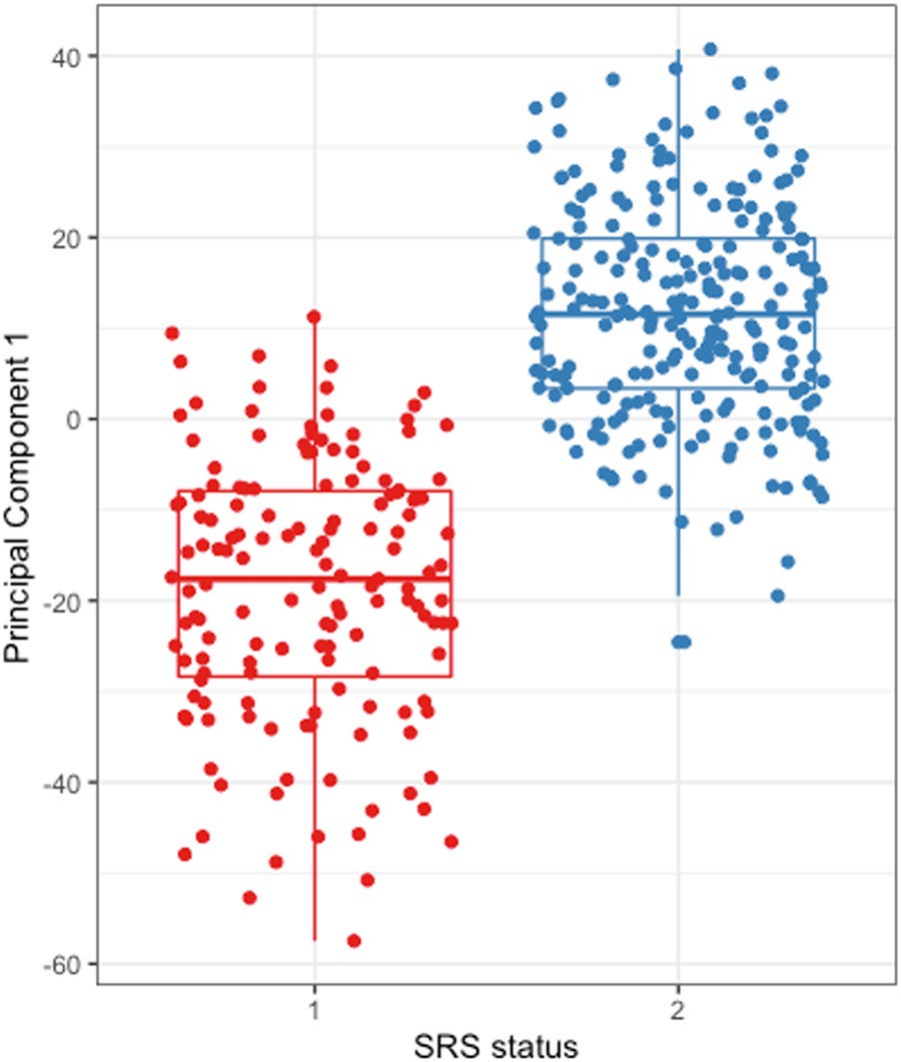
First principal component (PC1) from principal component analysis of the top 10% most variable genes expressed by peripheral blood leukocytes, plotted by Sepsis Response Signature (SRS) endotype from 390 patients with sepsis due to community-acquired pneumonia. Figure created using ggplot2 v3.2.0^34^.

SRS group membership (determined at the first available timepoint after ICU admission) was evaluated in the context of EBV positivity over the first five days of ICU admission. Considering SRS endotype as a categorical trait, there was a greater proportion of SRS1 patients within the EBV-positive group (44% vs 35%; p = 0.097) but this was not statistically significant (Supplementary Table 5). However, this binary classification is a simplification of what is a continuous spectrum of gene expression patterns. The SRS subgroups were originally identified through analysis of the expression of the top 10% most variable genes, with patients assigned to either SRS1 or SRS2 using a threshold value. The expression of these genes can be summarised through principal component analysis (PCA), showing that SRS group is strongly associated with principal component 1 (PC1) (Fig. 1), which accounts for 19.5% of the variance observed. This analysis illustrates the continuous nature of the SRS signature, with some individuals being found at either extreme of PC1, and others in the centre of the distribution with less pronounced differences in gene expression. We reasoned that considering SRS endotype as a continuous trait might increase statistical power to detect an association with EBV-positivity, as it would take into account the strength of the SRS-associated gene expression profiles. Thus, we compared PC1 between EBV-positive and EBV-negative individuals and found EBV-positive individuals had lower PC1 values, i.e. a more SRS1-like gene expression (mean PC1 score −2.8, s.d. 21.0 vs 1.8, s.d. 18.9; p = 0.014; t-test).

### Higher levels of EBV viraemia are seen in SRS1 patients

**Figure 2:**
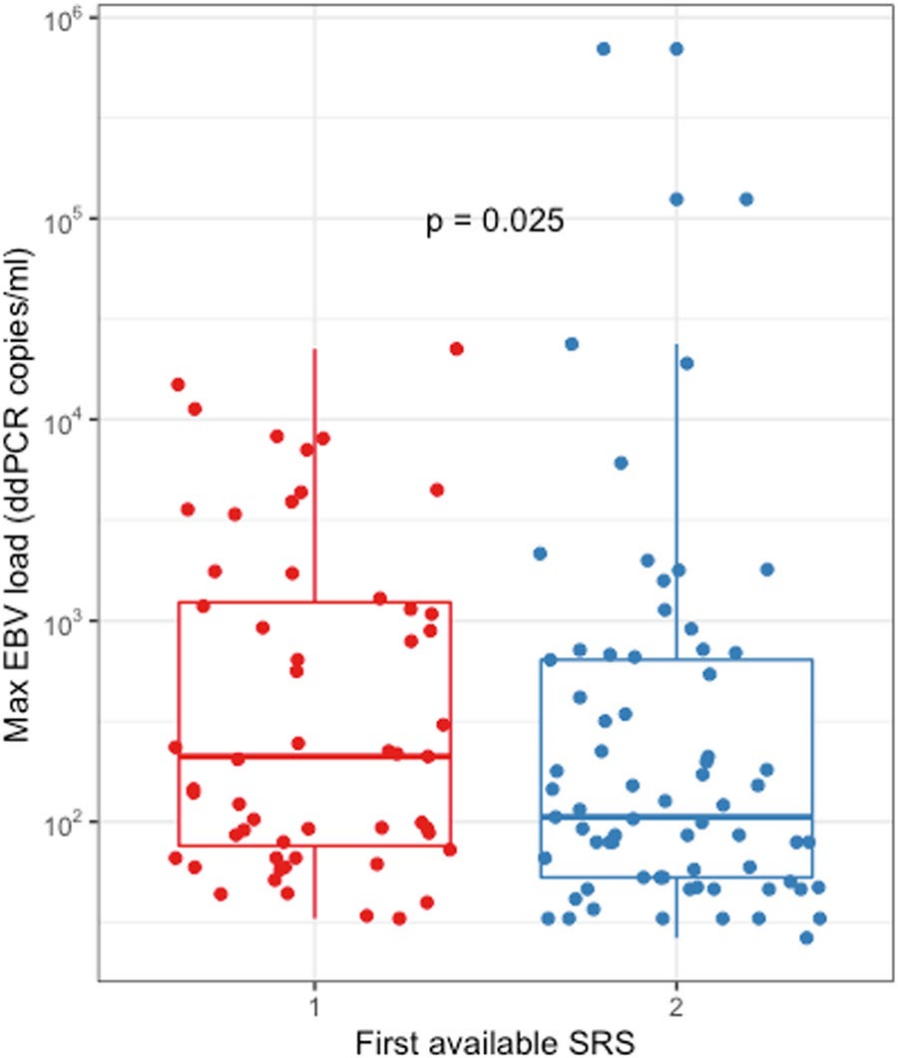
Maximum Epstein-Barr virus (EBV) load over the first 5 days of intensive care unit admission measured by digital droplet PCR (ddPCR), plotted by Sepsis Response Signature (SRS) status at the first available time point after intensive care unit admission. Figure created using ggplot2 v3.2.0^34^.

We hypothesised that if EBV positivity is associated with the SRS1 endotype, the two SRS endotypes might differ in the amount of EBV measured, among those in whom the virus was detected. Digital droplet PCR was used to assay EBV in plasma samples (619 samples; 565 patients) over the first five days of admission (days 1 and/or 3 and/or 5). The maximum EBV load was related to SRS endotype determined from the first available timepoint after ICU admission (Fig. 2). The median EBV load was higher in the SRS1 compared to the SRS2 endotype patients (211 vs 106 copies/ml; p = 0.025; Mann-Whitney U-Test).

### EBV viral load is associated with increased ICU length of stay

A linear model was fitted for each of the three morbidity measures with EBV viral load (as measured by ddPCR) and SRS as covariates. For ICU length of stay there was a significant association with both EBV viral load (p = 0.018) and SRS (p = 0.023). By contrast, there was no significant association between EBV viral load and day 1 SOFA score (p = 0.23) as well as maximum SOFA score (p = 0.17); SRS was associated with both day 1 SOFA score (p = 8.1 × 10^−7^) as well as maximum SOFA score (p = 4.1 × 10^−7^).

### EBV reactivation is associated with a distinct pattern of gene expression

**Figure 3:**
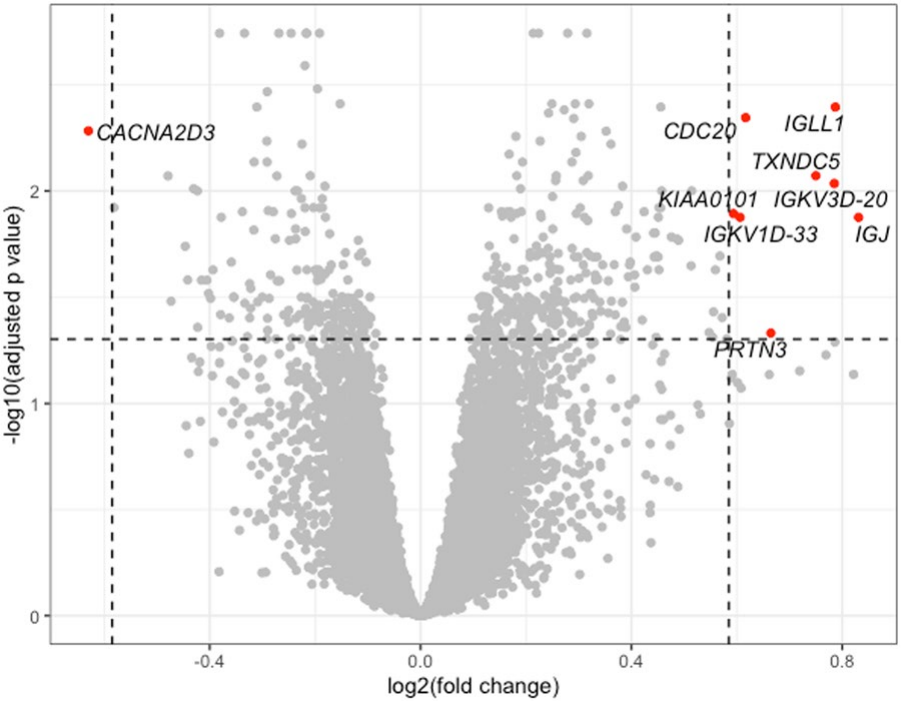
Volcano plot showing differentially expressed probes between Epstein-Barr virus-negative and Epstein-Barr virus-positive individuals. Labelled probes in red are differentially expressed at a fold change of 1.5 and false discovery rate of 0.05; positive fold change corresponds to upregulation in Epstein-Barr virus-positive individuals. Figure created using ggplot2 v3.2.0^34^.

Global gene expression from the peripheral blood total leukocyte population was compared between EBV-positive and EBV-negative individuals (Fig. 3). The differential expression analysis included only gene expression from samples at the first available time point; 28,228 probes were tested. Nine genes were differentially expressed at a fold change of 1.5 and FDR of 0.05 (Fig. 3). These include *CACNA2D3* (FDR 0.00521, fold change 1.55, downregulated in EBV-positive patients) which is a tumour suppressor gene downregulated in primary nasopharyngeal cancer and nasopharyngeal cancer cell lines compared with non-tumourigenic cells^15^ (EBV is implicated in the pathogenesis of nasopharyngeal cancer) and *CDC20* (FDR 0.00451, fold change 1.53, upregulated in EBV-positive patients) which binds to EBV-encoded proteins, activating the mitotic checkpoint and facilitating lytic EBV replication^16^. In addition, our analysis suggests a role for *PRTN3* (FDR 0.0468, fold change 1.59, upregulated in EBV-positive patients), which has been observed to be co-expressed with the basigin gene, overexpressed in nasopharyngeal cancer^17^ and *KIAA0101* (FDR 0.0128, fold change 1.51, upregulated in EBV-positive patients), an Epstein Barr virus Nuclear Antigen 1, EBNA-1 target gene^18^.

Since SRS status is associated with EBV status and is therefore a confounding factor, we repeated the differential expression analysis, this time including SRS as a covariate in the linear model (Supplementary Figure 1). Twelve genes were found to be differentially expressed; there was an overlap of seven genes with the previous analysis.

## Discussion

In this study, we aimed to establish the incidence of EBV reactivation in a large sepsis cohort, relate to outcome and clinical phenotypes, and test the hypothesis that viral reactivation in sepsis is related to host immunosuppression. To our knowledge, this is the first study to describe viral reactivation in sepsis in the context of subphenotyping patients using patient leukocyte transcriptomics to define sepsis endotypes that have been shown to be associated with outcome and treatment response. In the absence of a gold standard defining immunosuppression in sepsis, the SRS endotypes represent a proxy of immunosuppression that has clear clinical correlates.

We have shown that EBV reactivation in sepsis is common, increases over time and is associated with longer ICU stays and increased organ failure. Consistent with the concept that viral reactivation in sepsis is a consequence of immune compromise, EBV reactivation was associated with the SRS1 immunocompromised sepsis transcriptomic endotype. We also see differences in the molecular response to EBV reactivation in terms of total leukocyte gene expression profiles with a small number of differentially expressed genes (n = 9) between the EBV-positive and EBV-negative individuals. Although a number of these genes are of unknown function, *CACNAD3, CDC20, KIAA0101* and *PRTN3* are all implicated in EBV pathophysiology. Inclusion of SRS as a covariate is likely to have more effect on differentially expressed genes that were more specific to the degree of immunosuppression, reducing their significance and this may be the reason for the non-significance of *CACNA2D3* and *KIAA0101*. In clinical practice, viral reactivation in the critically ill is generally considered to be an epiphenomenon and a marker of illness severity that is of little clinical significance. It remains unclear whether reactivated viruses contribute to the pathophysiology of the host response to sepsis and whether specific treatment is indicated.

In previously published work, the SRS endotypes have been described as categorical traits^6^. Here we describe it for the first time as a continuous phenotype. By using the values of principal component 1, which separates patients with SRS1 from SRS2 in principal component analysis, we were able to show that individuals with a more pronounced SRS1 phenotype are more likely to develop reactivation of EBV, most likely as a consequence of decreased immune competence. One area for future work should therefore be to use immunophenotyping to determine whether higher levels of viraemia are associated with increased markers of immunosuppression.

Our observed association between EBV reactivation and morbidity highlights the clinical importance of this phenomenon. Although we observed an increase in 28-day mortality in individuals with EBV reactivation compared to those without (24% vs 16%; p = 0.051), this was on the borderline of statistical significance. This is in keeping with three large studies focusing on sepsis patients^8,9,12^ which used PCR to quantify EBV load. These studies did not find an association with EBV reactivation and mortality although one small study of critically ill sepsis and non-sepsis patients^19^ did find a positive association. We also found that EBV-positive patients had increased levels of organ dysfunction compared with EBV-negative patients. This is in keeping with the findings of Walton and colleagues^8^ as well as Mallet and colleagues^12^ who describe a similar association with SOFA score.

In our cohort (n = 573), we observed an overall 37% incidence of EBV reactivation. This finding is consistent with other studies in which the incidence of EBV reactivation in plasma has been estimated at 32–48%^8,9,12^. In contrast, we observed a lower rate of EBV and other viral reactivation in our metagenomic cohort. This is most likely because sample selection for metagenomics prioritised the sequencing of earlier time point samples to enable microbiological diagnosis of CAP with 40% of samples obtained on the first day of ICU admission. Walton and colleagues observed that the median time to reactivation for EBV was 5 days^8^ so it is highly probable that samples EBV-negative on day one would become EBV-positive at later timepoints. In addition, the comparatively low EBV viral loads observed are likely to represent the initiation rather than the peak of EBV viraemia in these individuals. Later assessment of viral reactivation beyond the first five days of ICU admission would have given further positive results for EBV and the other viruses documented in Table 2.

Our findings suggest some future directions to investigate, such as whether the levels of viraemia seen are actually detrimental and require treatment. The switch from latency to lytic replication requires the virus to escape from host immune surveillance and there is a possibility that EBV reactivation may compromise host immunity. For example, it is known that EBV reactivation is associated with increased expression of proteins such as an IL-10 homologue which inhibits monocyte/macrophage function^20^ and several proteins which impair interferon alpha and gamma release^21,22^. However, efforts to treat EBV viraemia will need to be carefully considered given that a trial of specific antiviral therapy in CMV reactivation had to be terminated early due to increased mortality in the treatment arm^23^.

Finally, immune therapies for sepsis have been and continue to undergo evaluation and it is likely that an individualised approach will be required. We propose that serial qPCR of EBV viral loads in the clinical setting be considered as part of a biomarker panel to comprehensively assess a patient’s immune status, such as that being used in the REAnimation Low Immune Status Markers (REALISM) project^24^.

## Conclusions

In critically ill patients with CAP and sepsis, EBV reactivation is associated with an immunosuppressed host transcriptomic endotype, greater degrees of organ dysfunction and prolonged ICU admission. These findings highlight the importance of immunosuppressive processes in the pathophysiology of sepsis.

## Methods

### Study design and participants

#### Recruitment

Adult patients admitted to intensive care with sepsis due to community-acquired pneumonia (CAP) were recruited through the UK Genomic Advances in Sepsis (GAinS) study (http://ukccggains.com) from 32 participating UK ICUs between 1 January 2005 and 31 December 2018. Patients were included on the basis of the 1992 ACCP/SCCM consensus definition of severe sepsis^25^ in use at study initiation. CAP was defined as a febrile illness with cough, sputum production, breathlessness, leucocytosis and radiological features of pneumonia acquired prior to or within 2 days of hospital admission^26^. Full inclusion and exclusion criteria are as previously described^6^.

#### Ethics approval and guidelines/regulations

Informed consent was obtained from all patients or their legal representative at the beginning of the study. This research was conducted under the following Research Ethics Committee approvals: 05/MRE00/38 (Scotland A Research Ethics Committee), 08/H0505/78 (Berkshire Research Ethics Committee). All methods were carried out in accordance with relevant guidelines and regulations.

### Procedures

#### Sample collection

Blood samples were collected into VACUETTE EDTA tubes (Greiner Bio-One, Kremsmünster, Austria) on the first and/or third and/or fifth day of ICU admission. A 5ml EDTA tube was centrifuged for 10 minutes at 4 °C and 1,600 g with resulting plasma stored long-term at −80 °C. For RNA, the total blood leukocyte population was isolated using LeukoLOCK filters (Life Technologies, Carlsbad, CA, USA) and stabilised using RNA*later* (Life Technologies), followed by total RNA extraction (Total RNA Isolation Protocol; Ambion, ThermoFisher Scientific, Waltham, MA).

#### Plasma nucleic acid extraction

Total nucleic acid extraction was performed using the NucliSENS easyMag platform (Biomerieux, Marcy-l’Étoile, France).

#### Metagenomics

Metagenomic sequencing was undertaken using the *Castanet* method described previously^14^. In brief, a combined RNA and DNA library preparation method was used followed by probe-based enrichment (Agilent SureSelect, Agilent, Santa Clara, CA, USA) using a custom RNA probe panel targeting bacteria and viruses relevant to paediatric meningitis and adult CAP. These included probes targeting 20 kb relatively low-diversity regions of EBV, CMV, human herpesvirus 6 (HHV-6) and herpes simplex viruses 1 and 2 (HSV-1 and HSV-2) as well as the full length of the JC virus genome. Sequencing was performed on the Illumina HiSeq 4000 or Illumina MiSeq generating 75 or 150 base paired-end reads. Data processing included classification of reads using Kraken v1^27^ and a custom database of human, viral, bacterial and fungal genomes followed by alignment of reads designated by Kraken as bacterial, viral or unclassified to consensus sequences corresponding to the probe targets using BWA v0.7.12^28^. A random forest classifier was then used to classify reads aligning to each organism in a sample as positive or negative.

#### Gene expression

Genome-wide gene expression data was generated using the Illumina Human-HT-12 v4 Expression BeadChip gene expression platform (47,231 probes; Illumina, San Diego, CA, USA). Four microarray datasets were generated (816 samples; 591 patients). Data preparation, background subtraction, and quality control were as previously described^6,29^. The four individual datasets were then combined with probe filtering, transformation, and normalisation using the vsn package^30^. The ComBat function from the R package sva^31^ was used to directly estimate and remove the known batch effects.

#### Digital droplet PCR (ddPCR)

Sample processing was performed in triplicate (1.5ul per replicate) following the recommended workflow (QX100 ddPCR system, Bio-Rad, Hercules, CA, USA). A custom PrimeTime primer/probe set (IDT DNA, Newark, NJ, USA) targeting a 78 bp amplicon of the EBV Epstein-Barr nuclear antigen 1 (*EBNA-1*) was designed based on published sequence data^32^ (Probe: 5′-/56-FAM/AGGGAGACA/ZEN/CATCTGGACCAGAAGGC/3IABkFQ/-3′; Forward primer: 5′-TACAGGACCTGGAAATGGCC-3′; Reverse primer: 5′-TCTTTGAGGTCCACTGCCG-3′). The reaction mixture was made up using ddPCR Supermix for Probes. A total of 20ul of each reaction mixture was loaded onto a DG8 cartridge with 70ul droplet generation oil and placed in the QX100 Droplet Generator (Bio-Rad). Droplets were transferred to a 96-well PCR plate and PCR amplification performed on a C1000 Touch Thermal Cycler (Bio-Rad). PCR conditions were as follows: 95 °C for 10 minutes; 40 cycles of 94 °C for 30 seconds, 60 °C for 1 minute; 98 °C for 10 minutes and hold at 4 °C. Following amplification, the plate was loaded onto the QX100 Droplet Reader (Bio-Rad) and data analysed with the QuantaSoft analysis software.

#### Serology

Enzyme-linked immunosorbent assay (ELISA) was used to test for the presence of IgG and IgM antibodies against the EBV viral capsid antigen (VCA; Abcam, Cambridge, UK) according to the manufacturer’s instructions. Plasma samples (10ul) were diluted to the recommended 1:100 concentration and run in duplicate. Absorbance was measured at 450 nm using the CLARIOstar plate reader (BMG Labtech, Ortenberg, Germany).

### Statistical analysis

Demographic and phenotypic data were compared using t-tests for continuous data, log-rank tests for mortality and length of stay data, Mann-Whitney tests for ordinal data and Chi-square tests for count data.

Differential gene expression was evaluated using the limma R package^33^, which fits a generalized linear model to the expression of each gene and employs an empirical Bayes approach to consider the overall variance in the data set. Genes with an FDR < 0.05 and a fold-change>1.5 were considered to be differentially expressed.

## Supplementary information

Supplementary Material.

## Data Availability

Gene expression data has previously been made available through the ArrayExpress database (accession numbers E-MTAB-4421/E-MTAB-4451/E-MTAB-5273/E-MTAB-5274). The metagenomics and digital droplet PCR datasets used during the current study are available from the corresponding author on reasonable request.
