# Supplementary material for: Epstein-Barr virus reactivation in sepsis due to community-acquired pneumonia is associated with increased morbidity and an immunosuppressed host transcriptomic endotype
Source: Sci Rep. 2020 Jun 17;10:9838. doi: 10.1038/s41598-020-66713-3 (PMC7299986; doi:10.1038/s41598-020-66713-3)

Supplementary Material: Epstein-Barr virus reactivation in sepsis due to community-acquired pneumonia is associated with increased morbidity and an immunosuppressed host transcriptomic endotype

Cyndi Goh[1], Katie L Burnham[2], M Azim Ansari[1,3], Mariateresa de Cesare[1,4], Tanya Golubchik[1,4], Paula Hutton[5], Lauren E Overend[1], Emma E Davenport[2], Charles J Hinds[6], Rory Bowden[1], Julian C Knight[1]*

[1]Wellcome Centre for Human Genetics, University of Oxford, Oxford, UK

[2]Wellcome Sanger Institute, Wellcome Genome Campus, Hinxton, UK

[3]Peter Medawar Building for Pathogen Research, University of Oxford, Oxford, UK

[4]Big Data Institute, University of Oxford, Oxford, UK

[5]Adult Intensive Care Unit, Oxford University Hospitals NHS Foundation Trust, Oxford, UK

[6]William Harvey Research Institute, Barts and The London School of Medicine, Queen Mary University, London, UK

***Corresponding author**

Prof Julian C Knight

julian@well.ox.ac.uk

Supplementary Table 1: Clinical characteristics and outcome data of ddPCR cohort. Epstein-Barr Virus-negative and Epstein-Barr Virus-positive individuals with ddPCR data are compared (total n=565). Intensive care unit length of stay analysis only includes patients surviving to intensive care discharge. Sepsis Response Signature endotype data was available on 314 patients. T-test performed except where indicated (^Chi-squared test; *Mann-Whitney U-test; §Log-rank test). Summary values refer to the mean. The range of values is provided where relevant.

**Characteristic**	**Epstein-Barr Virus-negative (n=328)**	**Epstein-Barr Virus-positive (n=237)**	**p-value**
Age (years)	60.5 (17-92)	62.3 (19-91)	0.18
Male sex^	200 (61%)	120 (51%)	0.018
Sepsis Response Signature 1^	71 (40%)	62 (45%)	0.60
Charlson Comorbidity Index*	2.6 (0-10)	2.8 (0-9)	0.41
ICU-acquired infection^	63 (19%)	57 (24%)	0.20
Mortality (28-day)§	56 (17%)	57 (24%)	0.05
Intensive care unit length of stay§ (days)	9.2 (1-101)	13.2 (1-55)	0.005
Sequential Organ Failure Assessment score (day 1)*	5.9 (0-17)	6.9 (1-19)	0.00048
Sequential Organ Failure Assessment score	6.7 (0-19)	8.0 (1-21)	2.1×10^{-5}

(maximum over first 7 days of admission)*			

Supplementary Table 2: Clinical characteristics and outcome data of metagenomics cohort. Epstein-Barr Virus-negative and Epstein-Barr Virus-positive individuals with metagenomics data are compared (total n=573). Intensive care unit length of stay analysis only includes patients surviving to intensive care discharge. Sepsis Response Signature endotype data was available on 311 patients. T-test performed except where indicated (^Chi-squared test; *Mann-Whitney U-test; §Log-rank test). Summary values refer to the mean. The range of values is provided where relevant.

**Characteristic**	**Epstein-Barr Virus-negative (n=383)**	**Epstein-Barr Virus-positive (n=190)**	**p-value**
Age (years)	59.9 (17-91)	63.8 (19-91)	0.0043
Male sex^	224 (58%)	100 (53%)	0.21
Sepsis Response Signature 1^	72 (36%)	51 (46%)	0.11
Charlson Comorbidity Index*	2.5 (0-10)	3.0 (0-9)	0.0075
ICU-acquired infection^	61 (16%)	41 (22%)	0.12
Mortality (28-day)§	64 (17%)	42 (22%)	0.10
Intensive care unit length of stay§ (days)	8.8 (1-75)	12.3 (1-51)	0.0040
Sequential Organ Failure Assessment score (day 1)*	5.8 (0-17)	7.1 (1-19)	1.9×10^{-5}

Sequential Organ Failure Assessment score (maximum over first 7 days of admission)*	6.6 (0-19)	8.0 (1-21)	1.2 x 10^{-5}

Supplementary Table 3: Epstein-Barr Virus status by day of sampling after intensive care unit admission. Plasma samples (n=1042) were collected on days 1 and/or 3 and/or 5 of ICU admission and EBV reactivation assessed by targeted metagenomics and/or ddPCR.

Day of sampling	EBV-negative	EBV-positive	Total
1	245 (75%)	82 (25%)	327
3	247 (70%)	106 (30%)	353
5	209 (58%)	153 (42%)	362

Supplementary Table 4: Microbiology of patient cohort. The microbiological causes of community-acquired pneumonia are compared between EBV-negative and EBV-positive individuals using a Chi-squared analysis. Patients with unknown microbiology are excluded from the statistical analysis.

Microbiology	EBV-negative	EBV-positive	p-value
Bacterial	115 (25%)	101(37%)	0.987
Viral	32 (7%)	27 (10%)	0.944
Mixed bacterial/viral	4 (1%)	5 (2%)	0.856
Fungal	0 (0%)	1 (0%)	0.952
Unknown	309 (67%)	137 (51%)	NA

Supplementary Table 5: Clinical characteristics and outcome data of gene expression cohort. Epstein-Barr Virus-negative and Epstein-Barr Virus-positive individuals with gene expression data are compared (total n=390). Intensive care unit length of stay analysis only includes patients surviving to intensive care discharge. T-test performed except where indicated (^Chi-squared test; *Mann-Whitney U-test; §Log-rank test). Summary values refer to the mean. The range of values is provided where relevant.

Characteristic	Epstein-Barr Virus-negative (n=239)	Epstein-Barr Virus-positive (n=152)	p-value
Age (years)	63.5 (19-91)	63.5 (17-91)	1.00
Male sex^	149 (62%)	88 (58%)	0.44
Sepsis Response Signature 1^	84 (35%)	67 (44%)	0.097
Charlson Comorbidity Index*	2.9 (0-10)	2.8 (0-9)	0.39
ICU-acquired infection^	51 (21%)	382 (25%)	0.48
Mortality (28-day)§	60 (25%)	48 (32%)	0.20
Intensive care unit length of stay§ (days)	10.7 (1-101)	13.4 (1-51)	0.070
Sequential Organ Failure Assessment score (day 1)*	6.2 (0-15)	7.0 (1-19)	0.019
Sequential Organ Failure Assessment score	7.1 (1-19)	8.1 (1-21)	0.0066

(maximum over first 7 days of admission)*			

Supplementary Figure 1: Volcano plot showing differentially expressed probes between Epstein-Barr Virus-negative and Epstein-Barr Virus-positive individuals with Sepsis Response Signature sepsis endotype included in the linear model. Labelled probes in red are differentially expressed at a fold change of 1.5 and false discovery rate of 0.05; positive fold change corresponds to upregulation in Epstein-Barr Virus-positive individuals. Figure created using ggplot2 v3.2.0 [34].

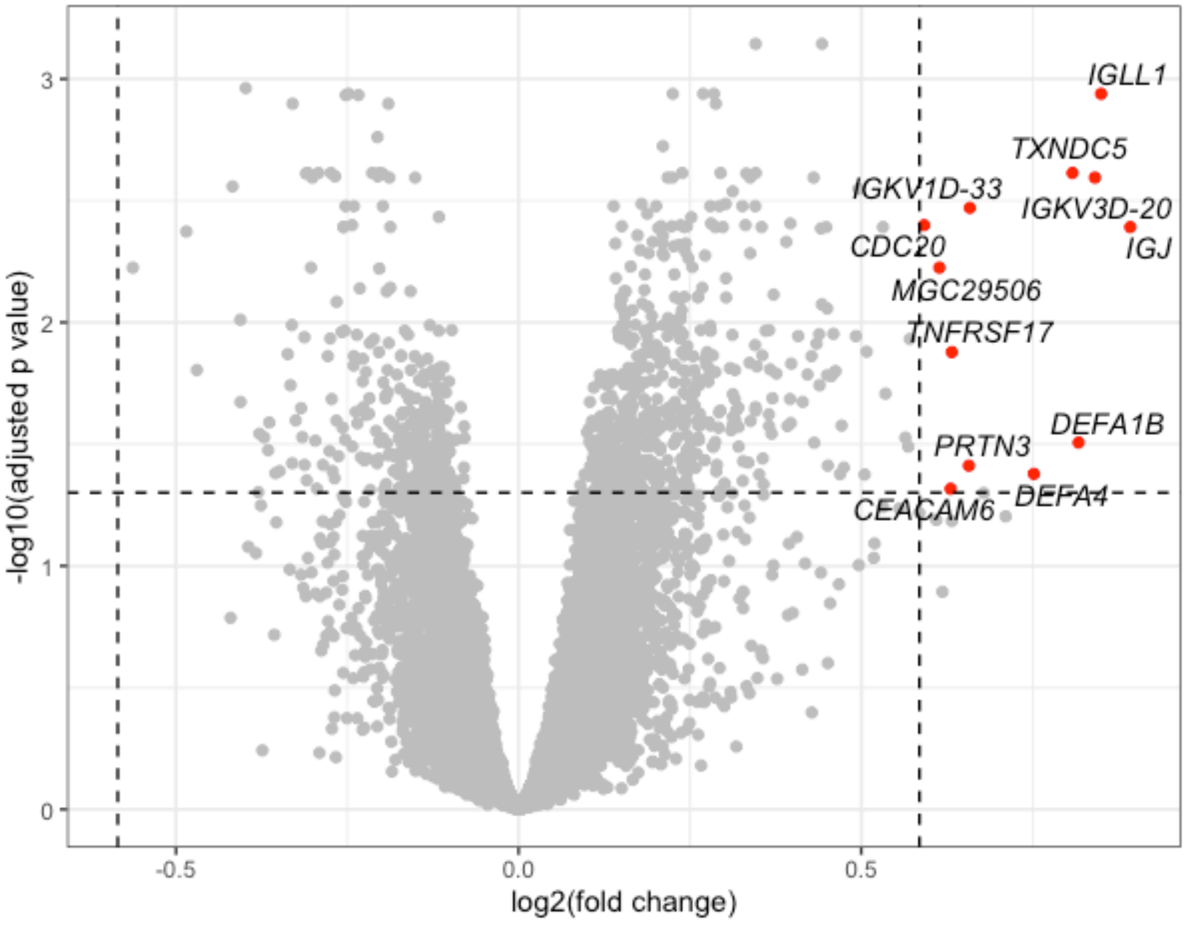

Supplement: Supplementary file 1 — Supplementary Material. [file 41598_2020_66713_MOESM1_ESM.pdf]
